# Vitamin D alleviates oxidative stress in varicose veins: a pilot study in obese and non-obese patients

**DOI:** 10.1007/s11010-025-05292-1

**Published:** 2025-04-22

**Authors:** Sonia Rațiu, Mihaela I. Mariș, Adina V. Furdui-Lința, Theia Stanciu-Lelcu, Claudia Borza, Sorin Olariu, Tiberiu Bratu, Adrian Sturza, Danina M. Muntean

**Affiliations:** 1 Doctoral School Medicine, “Victor Babeș” University of Medicine and Pharmacy of Timișoara, Timișoara, Romania; 2 Center for Translational Research and Systems Medicine, “Victor Babeș” University of Medicine and Pharmacy of Timișoara, Timișoara, Romania; 3 Department III – Chair of Pathophysiology, “Victor Babeș” University of Medicine and Pharmacy of Timișoara, Timișoara, Romania; 4 Department X – First University Clinic of Surgery, “Victor Babeș” University of Medicine and Pharmacy of Timișoara, Timișoara, Romania; 5 Department of Functional Sciences – Chair of Pathophysiology, Center for Translational Research and Systems Medicine, “Victor Babeș” University of Medicine and Pharmacy of Timişoara, 2, Eftimie Murgu Sq., 300041 Timişoara, Romania

**Keywords:** Chronic venous disease, Varicose veins, Obesity, Vitamin D deficiency, Oxidative stress, Antioxidant effect, Vitamin D receptor

## Abstract

Chronic venous disease and varicose veins of the lower extremities represent a widespread pathology, particularly in individuals with obesity. A high prevalence of varicose vein disease has been observed in obese patients in association with lower plasma levels of vitamin D. The present pilot study aimed to investigate the acute effects of 1,25-dihydroxyvitamin D_3_ [1,25(OH)_2_D_3_], the biologically active form of vitamin D, on oxidative stress in varicose veins obtained from both obese and non-obese patients undergoing cryostripping surgery for varicose vein ablation. Varicose venous samples treated or not with 1,25(OH)_2_D_3_ (100 nM, 12-h incubation) were analysed for reactive oxygen species (ROS) generation using the ferrous xylenol orange oxidation (FOX) assay and immunofluorescence technique. Additionally, the gene expression of endothelial nitric oxide synthase (eNOS), neuronal nitric oxide synthase (nNOS), and inducible nitric oxide synthase (iNOS) was assessed via qPCR. We report a significant reduction in circulating 25-hydroxyvitamin D_3_ [25(OH)D_3_] levels in obese as compared to non-obese patients. Ex vivo incubation of the venous samples with 1,25(OH)_2_D_3_ resulted in: (i) significant reduction in ROS level, (ii) upregulation of eNOS and nNOS expression, and (iii) downregulation of iNOS expression in both groups of patients. Vitamin D did not exhibit a ROS scavenger effect, and the antioxidant effect is presumably mediated via its receptor whose presence was confirmed in the varicose venous samples. In conclusion, vitamin D exerts protective effects in venous pathology, which may be beneficial in acute administration prior to the surgical intervention. Large clinical trials are required to assess the optimal dosage and time/duration of administration in patients with chronic venous disease with surgical indication.

## Introduction

Chronic venous disease (CVD) is a common vascular disorder characterized by impairment of the blood return in the venous circulation of the lower extremities with subsequent dilation and tortuosity of the superficial veins. The condition is primarily attributed to venous valve dysfunction, leading to venous hypertension, blood stasis, and the formation of varicose veins [1]. The prevalence of varicose veins is high, affecting approximately 20–30% of the adult population, with risk factors including age, female sex, pregnancy, prolonged standing, genetic predisposition, and obesity [2]. Despite their frequent occurrence, the underlying molecular mechanisms contributing to the pathogenesis of varicose veins remain incompletely understood, with oxidative stress and inflammation playing crucial roles in the pathophysiology of disease (recently reviewed in Refs. [3, 4]).

Oxidative stress has been widely recognized as a central pathophysiological mechanism involved in the development and progression of several vascular and rheumatic diseases [5–8] where the venous circulation becomes impaired. In the varicose veins, increased levels of reactive oxygen species (ROS) contribute to endothelial dysfunction, extracellular matrix remodelling, inflammation, and apoptosis, ultimately compromising venous wall integrity [9]. Several studies have demonstrated that ROS levels and the lipid peroxidation products, are significantly elevated in the varicose veins as compared to healthy veins [10, 11]. Furthermore, excessive ROS generation can disrupt nitric oxide (NO) homeostasis by reducing endothelial nitric oxide synthase (eNOS) activity, while upregulating inducible nitric oxide synthase (iNOS), leading to impaired vasodilation and exacerbated venous stasis [11].

Vitamin D deficiency has emerged as a critical factor in vascular health, with growing evidence suggesting a link between low serum vitamin D levels and increased oxidative stress, inflammation, and endothelial dysfunction [12]. Vitamin D, primarily in its active form 1,25-dihydroxyvitamin D_3_ [1,25(OH)_2_D_3_], exerts its biological effects through binding to the vitamin D receptor (VDR), which is expressed in various cell types, including endothelial cells and vascular smooth muscle cells [13, 14]. VDR activation has been shown to modulate oxidative stress by upregulating antioxidant enzymes such as superoxide dismutase (SOD) and catalase while downregulating pro-oxidant pathways [15, 16]. Additionally, vitamin D exerts anti-inflammatory effects by inhibiting nuclear factor-kappa B (NF-κB) signalling and reducing the production of inflammatory cytokines [17], which are known to contribute to the venous wall remodelling in the advanced stages of the disease. In the setting of diabetes, vitamin D administration has been reported to elicit systemic antioxidant and anti-inflammatory effects by increasing the level of reduced glutathione and reducing IL-8, respectively [18].

Obesity is a well-established risk factor for the varicose veins and is strongly associated with systemic inflammation, increased oxidative stress, and metabolic dysfunction [19]. Adipose tissue, in particular the visceral fat, is an active endocrine organ that secretes pro-inflammatory cytokines such as tumour necrosis factor-alpha (TNF-α) and interleukin-6 (IL-6), both of which have been implicated in endothelial dysfunction and venous wall degeneration [20, 21]. Moreover, obesity is frequently linked to vitamin D deficiency due to sequestration of the fat-soluble vitamin in adipose tissue, leading to its reduced bioavailability [22]. This deficiency further exacerbates oxidative stress and inflammation creating a vicious cycle that may contribute to the increased cardiovascular risk, morbidity and mortality [23].

Despite the described interplay among oxidative stress, vitamin D deficiency, and obesity, the investigation of the potential therapeutic role of vitamin D in modulating redox homeostasis in the varicose veins has been less addressed in the literature.

The present pilot study was purported to assess the in vitro effects of 1,25(OH)_2_D_3_, the biologically active form of vitamin D, on oxidative stress in varicose veins harvested from both obese and non-obese patients undergoing cryostripping surgery for the varicose veins ablation.

## Materials and methods

### Characteristics of the study groups

Varicose vein samples were collected from 31 consecutive patients with varicose veins and indication for surgery hospitalized at the First University Clinic of Surgery, “Pius Brînzeu” Emergency County Hospital, Timișoara, Romania. Venous samples harvested from 3 out of the 31 patients were discarded due to subjectively presumed impaired sample quality (inappropriate collection/transport). A total number of 28 patients were assigned to one of two groups based on their body mass index (BMI): (1) obese patients (OB, *n* = 12) with BMI ≥ 30 kg/m^2^ and (2) non-obese patients (NON-OB, *n* = 16) with BMI < 30 kg/m^2^. The study protocol was reviewed and approved by the university Ethics Committee for Scientific Research (no. 62/17.12.2020). In compliance with ethical guidelines, all participants provided written informed consent before the intervention, according to the principles outlined in the World Medical Association’s Declaration of Helsinki.

Characteristics of the study groups, including demographic data, and relevant biochemical parameters are summarized in Table 1. Additionally, information regarding their comorbidities and medication is detailed in Table 2.

**Table 1:** Characteristics of the patients included in the study

Parameter	OB (*n* = 12; 7 ♀, 5 ♂)	non-OB (*n* = 16; 15 ♀, 1 ♂)	*p*
**BMI (kg/m**^**2**^**)**	**34.40 ± 1.45**	**25.92 ± 0.67**	**< 0.05**
Age (y)	55.66 ± 3.51	52.94 ± 3.19	ns
**25(OH)D**_**3**_	**19.93 ± 1.93**	**22.26 ± 2.51**	**< 0.05**
Blood count			
RBC (mil/mm^3^)	5.06 ± 0.11	4.44 ± 0.08	ns
Ht (%)	44.16 ± 0.78	38.82 ± 0.91	ns
Hb (g/dL)	14.38 ± 0.22	12.78 ± 0.37	ns
WBC (× 10^3^/mm^3^)	7760.83 ± 588.63	6388.55 ± 639.97	ns
PLT (/mm^3^)	209 583.33 ± 9523.86	277 411.76 ± 19,957.13	ns
ESR (mm/h)	14.4 ± 1.85	15 ± 3.31	ns
**C-reactive protein (mg/L)**	**5.55 ± 1.29**	**2.38 ± 0.76**	**< 0.05**
Urea (mg/dL)	34.63 ± 3.88	33.76 ± 2.51	ns
Creatinine (mg/dL)	0.83 ± 0.05	0.81 ± 0.04	ns
Uric acid (mg/dL)	5.22 ± 0.41	4.51 ± 0.31	ns
**Total cholesterol (mg/dL)**	**175.66 ± 10.58**	**207.41 ± 7.27**	**< 0.05**
HDLc (mg/dL)	56.66 ± 7.40	65.70 ± 4.59	ns
**LDLc (mg/dL)**	**99.83 ± 9.77**	**124.12 ± 6.34**	**< 0.05**
Triglycerides (mg/dL)	115.58 ± 18.40	96.94 ± 14.55	ns
Blood glucose (fasted) (mg/dL)	109.83 ± 7.10	99.58 ± 5.55	ns
**ALAT (U/L)**	**35.5 ± 6.44**	**24.31 ± 2.05**	**< 0.05**
**ASAT (U/L)**	**24.33 ± 2.77**	**18.88 ± 1.54**	**< 0.05**
Total serum calcium (mg/dL)	9.25 ± 0.16	9.21 ± 0.12	ns
Ionized calcium (mg/dL)	3.94 ± 0.06	3.98 ± 0.05	ns
Serum phosphate (mg/dL)	3.45 ± 0.23	3.84 ± 0.14	ns

Parameters in bold have statistical significance when comparing groups

**Table 2:** Comorbidities and medication of patients included in the study

	OB	non-OB
CVD + comorbidities	CVD CEAP C2-C3, Thrombophlebitis, Hypertension, Diabetes, Dyslipidemia, Hypothyroidism, Hepatitis B, Hyperuricemia, Prostate adenoma	CVD CEAP C2-C3, Thrombophlebitis, Hypertension, Diabetes, Dyslipidemia, Hypothyroidism, Asthma, Hypoacusia, Glaucoma, Uterine fibroma, Osteoporosis
Medication	Nebivolol, Bisoprolol, Metoprolol, Indapamid, Zofenopril, Quinapril, Candesartan, Aspirin, Enoxaparin, Atorvastatin, Rosuvastatin, Sulodexide, Detralex, Metformin, Insulin, Gliclazide, Dulaglutide, Levothyroxine, Entecavir, Allopurinol, Benfotiamine, Cholecalciferol, Magnesium orotate	Metoprolol, Indapamide, Amlodipine, Perindopril, Sulodexid, Candesartan, Aspirin, Acenocumarol, Detralex, Metformin, Levothyroxine, Saleterol, Fluticasone, Betahistin, Dydrogesterone, Alendronic acid, Cholecalciferol, Omega 3, Vitamin A, Vitamin E, Vitamin B6, Bupropion

### Experimental procedure

After surgery, the varicose veins samples collected in Hanks’ solution were immediately placed on ice and transported to the laboratories of the Center for Translational Research and Systems Medicine where they were carefully cleaned and prepared for further analysis. The samples were then incubated at 37 °C in endothelial cell growth basal medium (EBM) containing 0.1% bovine serum albumin (BSA), in the presence or absence of the active form of vitamin D, 1,25(OH)_2_D_3_ (100 nM), for a duration of 12 h. The concentration of 100 nM for vitamin D was selected based on previous studies demonstrating its ex vivo beneficial effects on animal vascular and smooth muscle cells [24, 25] and also, on human mesenteric arteries [26].

### Oxidative stress assessment by ferrous iron xylenol orange oxidation assay

To evaluate hydrogen peroxide (H_2_O_2_) production, we used the Ferrous Iron Xylenol Orange Oxidation (FOX) assay (PeroxiDetect Kit, Merck Sigma-Aldrich), following established protocols [27, 28]. This assay operates on the principle that peroxides oxidize ferrous ions (Fe^2+^) to ferric ions (Fe^3+^) under acidic conditions. The Fe^3+^ ions then form a coloured complex with xylenol orange, which can be quantitatively measured by spectrophotometry at a wavelength of 560 nm. Using a standard curve, we calculated the hydrogen peroxide production, and the results were expressed as nmol H_2_O_2_ per hour per mg of tissue.

### Oxidative stress assessment by immuno-fluorescence

ROS levels were also assessed using the dihydroethidium (DHE) probe, according to previously published protocols [28, 29]. This method allows for the detection of superoxide anion production within tissue sections, providing valuable insight into oxidative stress levels. For sample preparation, varicose vein tissues were embedded in optimal cutting temperature (OCT) compound and rapidly snap-frozen to preserve tissue integrity. The frozen specimens were then sectioned into 20 µm thick cryosections and mounted onto glass slides. To ensure optimal staining conditions, the slides were subjected to three consecutive washes with phosphate-buffered saline (PBS), each lasting five minutes. The cryosections were subsequently incubated with DHE in a dark environment at room temperature for 30 min to allow for ROS-dependent fluorescence development. Following incubation, excess DHE was carefully removed through an additional series of three PBS washes. The slides were then mounted using Vectashield antifade mounting medium (Vector Laboratories) to preserve fluorescence signal intensity and prevent photobleaching. Imaging and analysis were performed immediately using an Olympus Fluoview FV1000 confocal microscope. Fluorescent signals were captured using laser excitation at 488 nm, enabling the visualization and quantification of ROS within the tissue samples.

### Vitamin D receptor staining in immunofluorescence

The expression of the vitamin D receptor (VDR) in human varicose veins was evaluated using immunofluorescence analysis on frozen tissue sections. To detect VDR, we utilized a specific primary antibody (Abcam, ab109234, 1:50 dilution), followed by incubation with an Alexa Fluor-conjugated secondary goat anti-rabbit antibody (Invitrogen, A32731, 1:200 dilution). To visualize cell nuclei, we applied 4′,6-diamidino-2-phenylindole (DAPI) staining (Santa Cruz, SC3598), which allowed for clear identification of nuclear localization within the tissue sections. This protocol was carried out in accordance with previously established methodologies [27, 28]. Imaging and analysis were performed using an Olympus Fluoview FV1000 confocal microscope, ensuring high-resolution visualization of VDR expression within the VV samples.

### NOS expression assessment by qPCR

Tissue samples were homogenized using the TissueLyser system (Qiagen) to ensure efficient cell disruption and RNA extraction. Total RNA was then isolated using the Total RNA Mini SI Isolation Spin-Kit (Applichem), and its concentration and purity were assessed with a Nanodrop 2000 spectrophotometer (Thermo Scientific). The extracted RNA was subsequently used for complementary DNA (cDNA) synthesis through reverse transcription, performed with the Superscript III RT kit (Invitrogen). Quantitative real-time PCR (qRT-PCR) was conducted using a Bio-Rad system (CFX Connect Real-Time PCR Detection System) to evaluate gene expression levels in the venous samples. Specific primers targeting the three nitric oxide synthase (NOS) isoforms were used for amplification: endothelial nitric oxide synthase (eNOS), Forward-CTG CTG CCC GAG ATA TCT TC, Reverse-CAG GTA CTG CAG TCC CTC CT; inducible nitric oxide synthase (iNOS), Forward:-CTT TGG CCT GTC CGG TTC CC, Reverse-TGG GGC AGT CTC CAT TGC CA; neuronal nitric oxide synthase (nNOS), Forward: GGC ACT GGC ATC GCA CCC TT, Reverse:-CTT TGG CCT GTC CGG TTC CC. To normalize gene expression, the housekeeping gene eukaryotic elongation factor 2 (EEF2) was used as an internal control with the following primers: Forward- GAC ATC ACA AGG GTG TGC AG, Reverse- GCG GTC AGC ACA CTG GCA TA.

### Statistics

Data are expressed as mean ± standard error of the mean (SEM) to provide a measure of variability within each group. Normality was assessed using the Shapiro–Wilk test before applying parametric analyses. For direct comparisons between two groups, the Student *t*-test was applied. One-way ANOVA was employed when comparing multiple groups, followed by post-hoc Tukey test when necessary to identify specific group differences. A *p*-value of less than 0.05 (*p* < 0.05) was considered the threshold for statistical significance. Statistical analysis was performed using GraphPad Prism software (v. 9.3.1, GraphPad, USA).

## Results

### Obesity is associated with low plasma levels of vitamin D

**Fig. 1:**
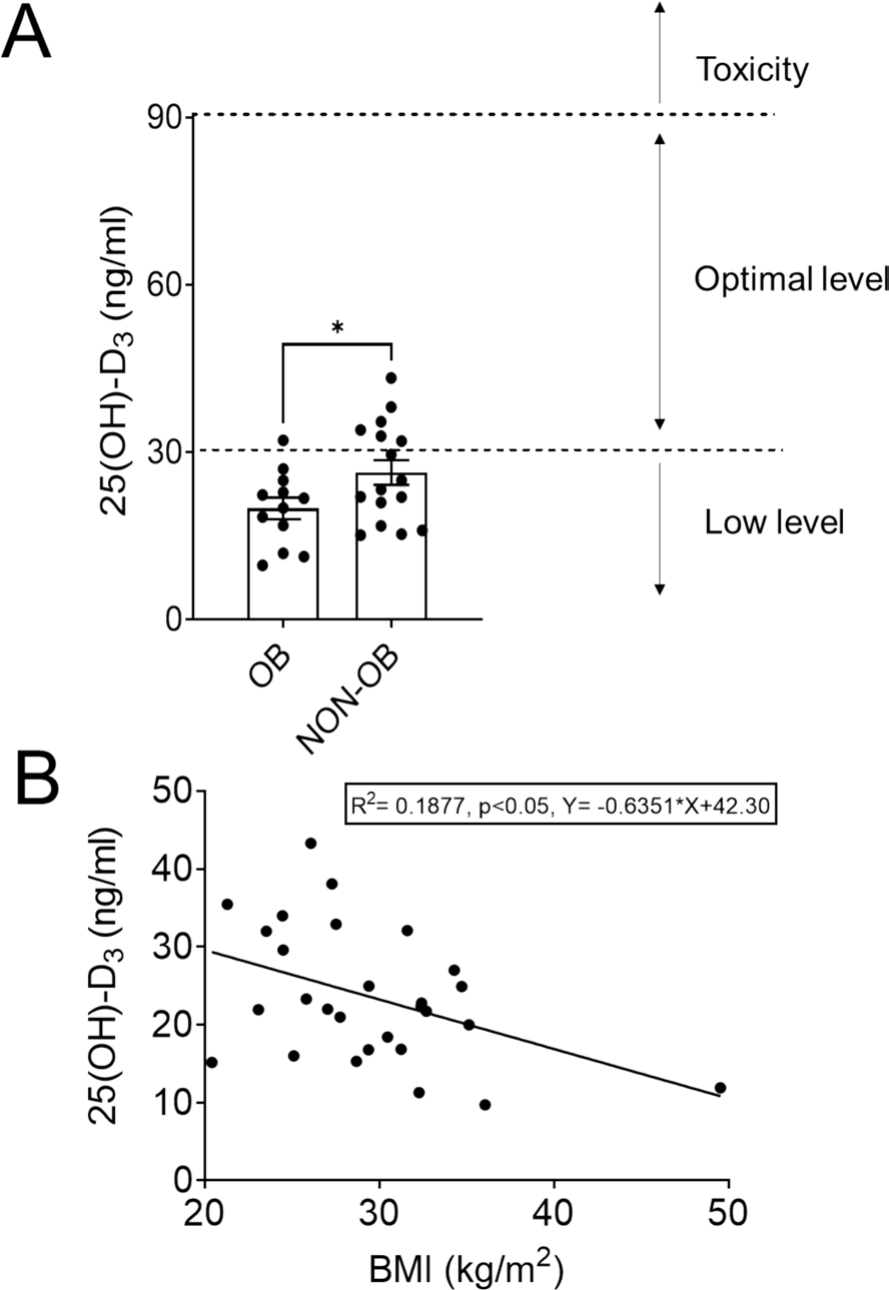
Obesity is associated with low plasmatic levels of vitamin D. **A** Level of 25(OH)D_3_ in obese (OB, *n* = 12) and non-obese (NON-OB, *n* = 16) groups. **B** Correlation between 25(OH)D_3_ level and body mass index (BMI). *p < 0.05

Plasma concentration of 25(OH)D_3_ was measured as indicator of the vitamin D status. Our findings revealed that all participants, regardless of obesity status, had vitamin D levels below the optimal threshold (Fig. 1A). However, a statistically significant difference was observed between obese and non-obese patients, with the obese group exhibiting lower 25(OH)D_3_ levels (Fig. 1A). Additionally, we identified a positive correlation between plasma 25(OH)D_3_ concentration and obesity level, suggesting a potential link between vitamin D deficiency and BMI (Fig. 1B).

Furthermore, the Ob group with vitamin D deficiency also showed higher levels of C-reactive protein (CRP) vs the NON-OB one (*p* < 0.05), indicative of low-grade systemic inflammation (Table 1).

### In vitro incubation with the active vitamin D mitigated venous oxidative stress

Given the low serum vitamin D in both study groups (particularly in the obese group, which also showed increased inflammation), we sought to explore whether acute exposure of the varicose vein samples to the active form of vitamin D, 1,25(OH)_2_D_3_ (calcitriol) at a concentration of 100 nM, could modulate the local oxidative stress. To this aim, we measured the hydrogen peroxide (H_2_O_2_) level by means of FOX assay and the superoxide (O_2_^**·**−^) via immunofluorescence (IF).

**Fig. 2:**
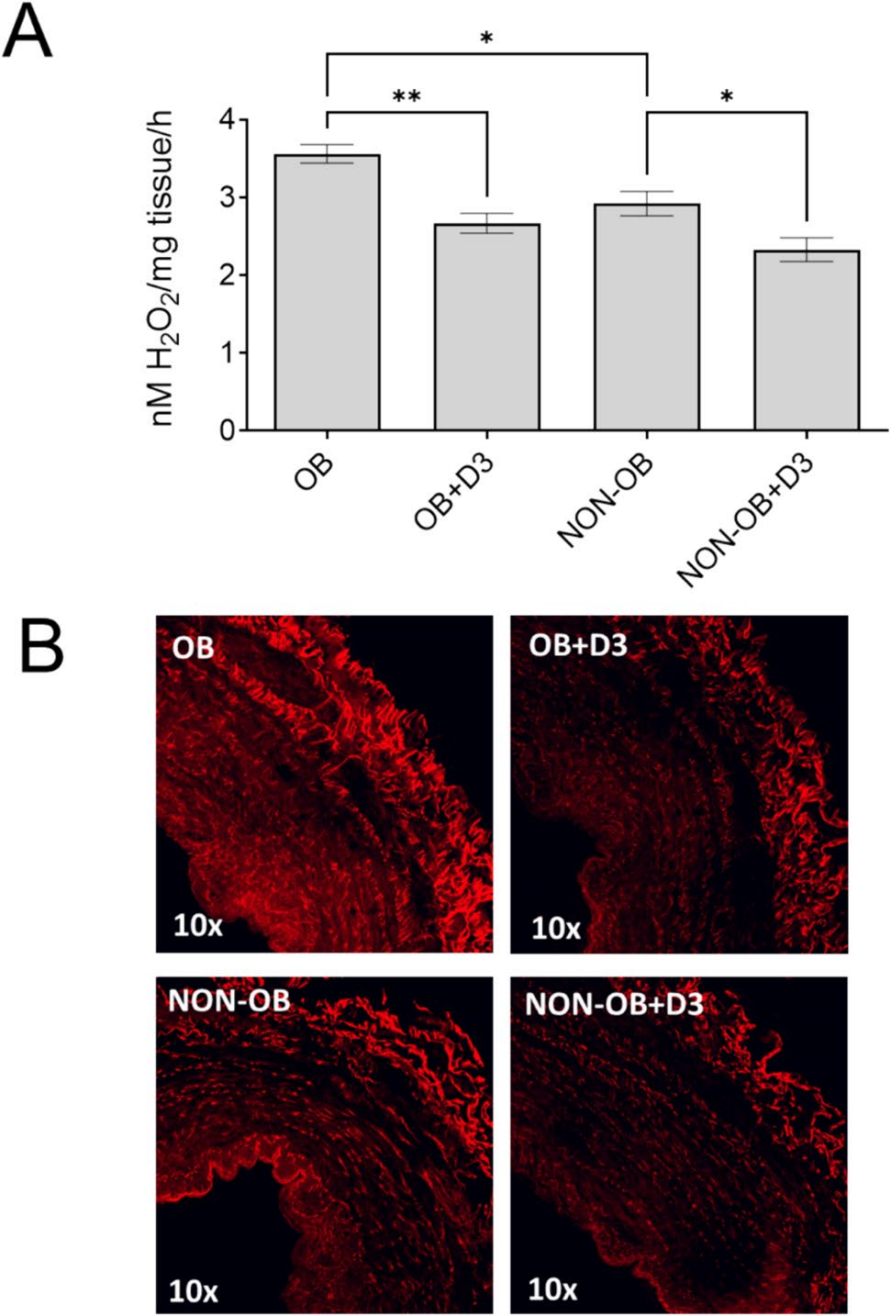
The active form of vitamin D – 1,25(OH)_2_D_3_ reduces oxidative stress in the varicose veins. **A** FOX assay. **B** DHE staining. *p < 0.05, **p < 0.01. *OB* obese, *n* = 12; *NON-OB* non-obese, *n* = 16

Our findings, as illustrated in Fig. 2, indicate that oxidative stress was significantly elevated in the obese group compared to the non-obese group. However, after a 12-h incubation period with active vitamin D, there was a noticeable reduction in H_2_O_2_ levels detected by the FOX assay (Fig. 2A), as well as a decrease in O_2_^**·**−^ level, as evidenced by IF analysis (Fig. 2B). These results suggest that vitamin D exerts a protective antioxidant effect on varicose veins. Notably, this beneficial effect was observed in both obese and non-obese groups, underscoring the potential role of vitamin D in improving the oxidative status of the venous bed across different patient populations.

### Vitamin D modulated the expression of NOS isoforms

In order to understand the mechanisms underlying the beneficial antioxidant effects of vitamin D in human venous samples we investigated whether it modulates the expression of the enzymes involved in nitric oxide (NO) synthesis, as NO plays a crucial role in maintaining vascular health by promoting vasodilation and protection against endothelial dysfunction [30].

**Fig. 3:**
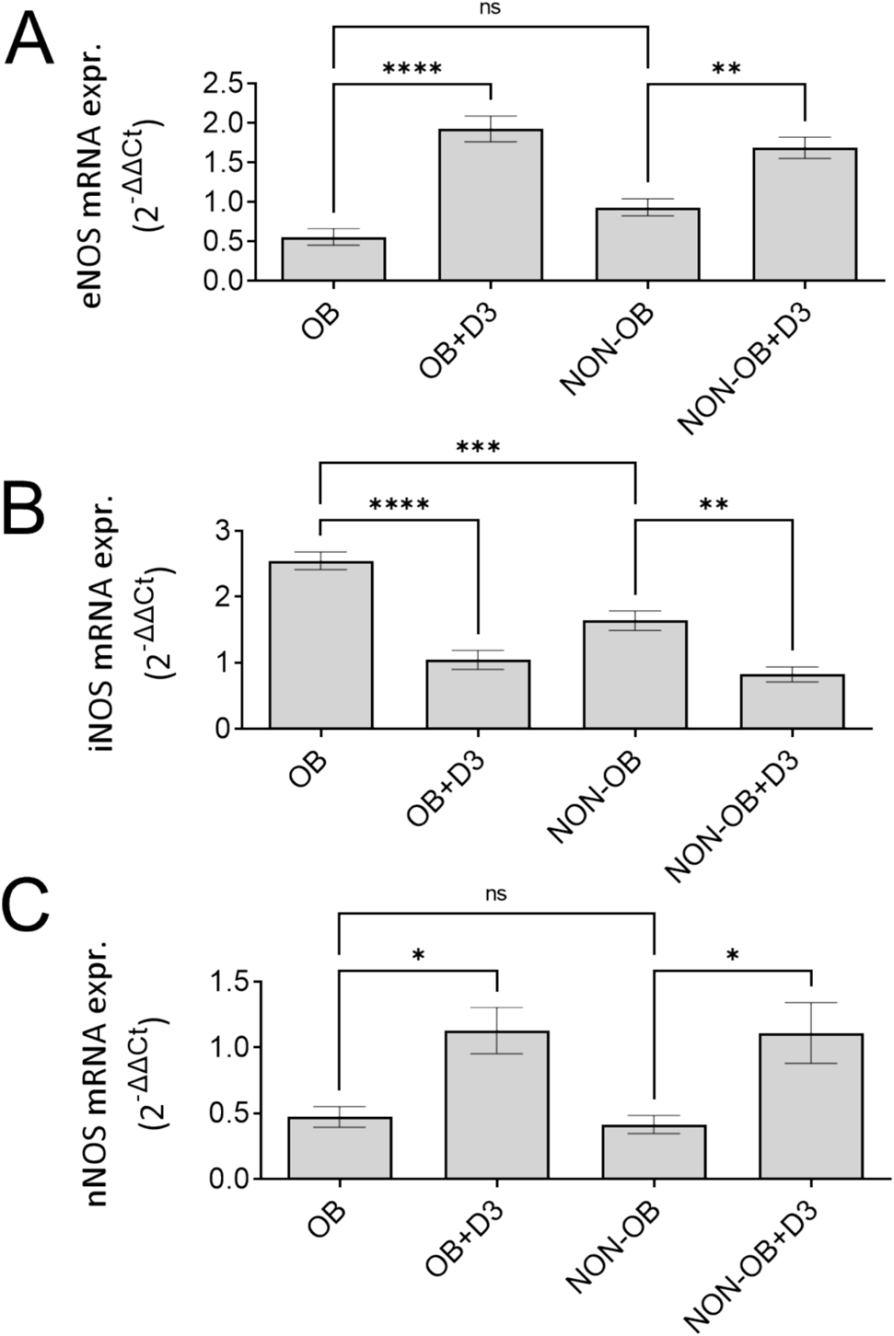
The effect of 1,25(OH)_2_D_3_ on the mRNA expression of the NOS isoforms in varicose veins samples from obese (OB, *n* = 12) and non-obese (NON-OB, *n* = 16) patients. **A** eNOS—endothelial nitric oxide synthase, **B** iNOS—inflammatory nitric oxide synthase, **C** nNOS—neuronal nitric oxide synthase. *p < 0.05, **p < 0.01, ***p < 0.001, ****p < 0.001

The gene expression of the three nitric oxide synthase (NOS) isoforms was investigated: endothelial NOS (eNOS), inducible NOS (iNOS), and neuronal NOS (nNOS) following in vitro stimulation with the active form of vitamin D, 1,25(OH)_2_D_3_. Acute incubation with the active form of vitamin D, 1,25(OH)_2_D_3_ significantly upregulated eNOS and nNOS with the concomitant downregulation of iNOS gene expression (Fig. 3). No significant differences were observed for eNOS and nNOS levels between obese and non-obese individuals. However, iNOS expression was markedly elevated in the obese group, suggesting a heightened inflammatory state that may contribute to vascular dysfunction in obesity-associated varicose veins.

These findings suggest that vitamin D may contribute to venous protection by enhancing NO bioavailability through eNOS upregulation while simultaneously reducing the inflammation-associated NO production via iNOS suppression.

### The active vitamin D had no scavenger effect for hydrogen peroxide

In order to further dissect the beneficial antioxidant effect of vitamin D on the varicose veins, we wondered whether vitamin D exhibits scavenging properties for hydrogen peroxide, similar to the enzymatic antioxidants. To determine whether vitamin D directly neutralizes H_2_O_2_, we compared its effects to the one of catalase, the classic antioxidant, by assessing the % of H_2_O_2_ (100 µM) neutralization in the presence of increasing concentrations of vitamin D (1 nM, 100 nM, 10 μM).

**Fig. 4:**
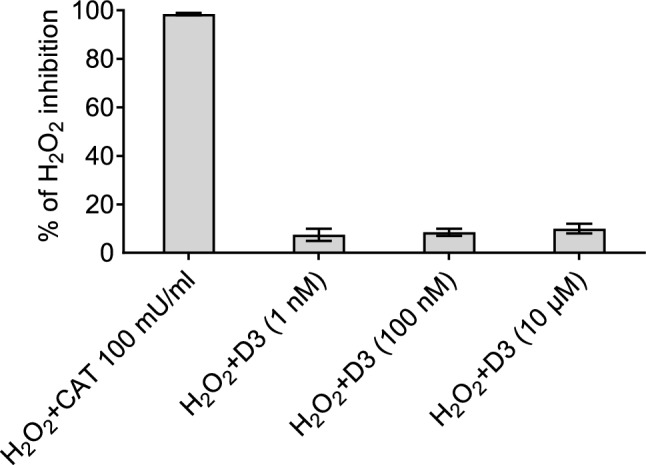
The effect of vitamin D. FOX assay for reduction of H_2_O_2_ level in the presence of different concentrations of 1,25(OH)_2_D_3_ *vs* catalase (100 mU/mL), the classic ROS scavenger

As shown in Fig. 4, the active vitamin D, unlike catalase, did not exhibit direct scavenging properties for H_2_O_2_. This suggests that its antioxidant effect observed in varicose veins was not due to the direct neutralization of ROS.

### Vitamin D receptor is expressed at the level of varicose veins

**Fig. 5:**
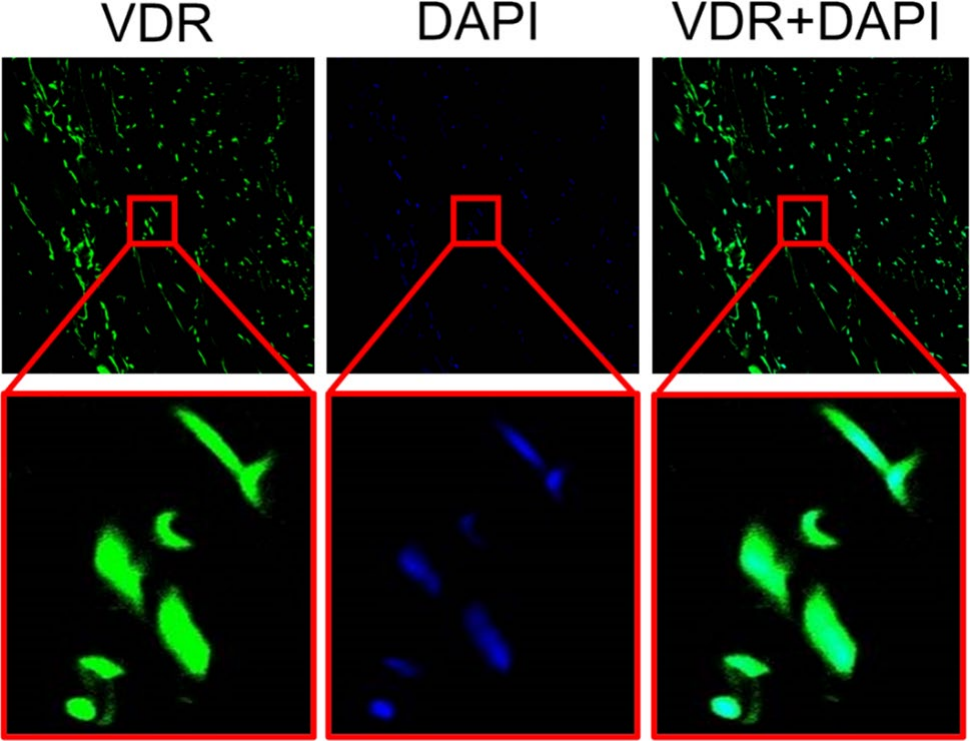
Expression of vitamin D receptor (VDR) in the varicose veins. VDR (green), DAPI (blue)

Last but not least, we assessed the vitamin D receptor (VDR) expression in the varicose veins by means of immunofluorescence (Fig. 5). We showed the nuclear presence of the VDR (colocalization with DAPI), which suggests that varicose veins have the capacity to respond to vitamin D signalling.

This finding indirectly suggests that the beneficial effects of vitamin D are mediated through its interactions with the VDR (since no direct chemical neutralization of ROS was observed).

## Discussions

The major finding of this study carried out on varicose vein samples harvested from obese and non-obese patients undergoing cryostripping surgery is that in vitro incubation with the active vitamin D mitigated the venous oxidative stress in varicose veins and modulated the expression of nitric oxide synthase isoforms. These effects were presumably mediated through its interaction with the vitamin D receptor whose presence was detected in the samples. Additionally, obese patients with pronounced vitamin D deficiency exhibited elevated levels of C-reactive protein (CRP), as indicator of the associated low-grade systemic inflammation.

A plethora of studies reported the link between vitamin D deficiency and the development of cardiovascular diseases and/or their complications (recently reviewed in Refs. [31–33]). However, research on how vitamin D deficiency is connected with the chronic venous disease progression is rather scarce [34–36]. Vitamin D plays a vital role in preserving vascular integrity by regulating endothelial function and mitigating oxidative stress [14, 37, 38]; low levels of vitamin D may contribute to the increase in vascular permeability, venous wall remodelling, and possibly, also the valvular dysfunction. Additionally, chronic venous disease (CVD) is linked to the combination between persistent inflammation and oxidative stress [39, 40], both being responsible for the progressive venous wall degradation and remodelling. Vitamin D exerts anti-inflammatory and antioxidant effects by reducing the level of pro-inflammatory cytokines and lowering ROS production [4, 17, 41]. Furthermore, obesity, the most important risk factor for both vitamin D deficiency and CVD, is associated with subclinical inflammation, insulin resistance, and elevated venous pressure [42, 43]. The fact that low vitamin D levels in obese individuals may further exacerbate venous dysfunction by impairing nitric oxide bioavailability and increasing oxidative stress has been reported in the literature [17, 38, 44], but no studies investigated, to the best of our knowledge, the NOS isoforms local expression in the varicose veins.

Importantly, our finding that VDR is expressed in the varicose veins aligns with previous research demonstrating the presence of VDR in various vascular territories [45, 46]. Previous research has established that VDR is expressed in endothelial cells, vascular smooth muscle cells, and macrophages within the vascular wall being involved in involved in vascular homeostasis [13, 47]. Studies have shown that VDR activation plays a critical role in regulating vascular tone, endothelial function, and inflammatory responses [48]. In the arterial wall it was demonstrated that VDR activation enhanced eNOS activity, improved vascular relaxation and decreased oxidative stress [49]. However, studies investigating VDR expression specifically in varicose veins are relatively limited. Since endothelial dysfunction is also present in the varicose veins, the finding of VDR venous expression suggests that vitamin D may influence the vein function and/or remodelling in this widespread pathology. We have to acknowledge as a limitation of the present study the fact that we did not assess the VDR expression in healthy venous samples. If VDR is differentially regulated in varicose veins as compared to normal veins, this could provide insights into its role in disease progression.

A number of studies reported that VDR expression can be altered under pathological conditions such as atherosclerosis, where inflammation and oxidative stress also negatively impact on vitamin D signalling [50, 51]. Other studies reported the immunomodulatory role of vitamin D in the setting of inflammatory diseases, emphasizing its ability to suppress pro-inflammatory cytokine production in lungs and intestines [52, 53]. Whether vitamin D signalling plays a role in the inflammation underlying the varicose vein pathology remains an open research area.

Oxidative stress arises from an imbalance between increased ROS production and decreased antioxidant defense, contributing to endothelial dysfunction in the setting of vascular diseases. The main enzymatic sources of ROS are: the dysfunctional mitochondrial respiratory chain, NADPH oxidases (NOX), xanthine oxidase (XO), and monoamine oxidase (MAO). Emerging evidence suggests that vitamin D, particularly in its active form [1,25(OH)_2_D_3_], plays a regulatory role in modulating these oxidative pathways, thereby exerting protective effects against oxidative damage. Vitamin D has been shown to suppress NOX expression and activity, particularly NOX2 and NOX4 isoforms, in endothelial and vascular smooth muscle cells; this inhibition reduces ROS generation, prevents oxidative damage and endothelial dysfunction, as observed in hypertension, atherosclerosis, and diabetes [54–57]. Furthermore, vitamin D enhances mitochondrial function by improving oxidative phosphorylation efficiency, reducing electron leakage, and upregulating antioxidant enzymes, such as superoxide dismutase (SOD) and glutathione peroxidase (GPx). This contributes to reduced mitochondrial ROS production and improved cellular bioenergetics [58, 59]. Also, vitamin D has been reported to downregulate XO activity, reducing oxidative stress and endothelial dysfunction, thereby mitigating vascular inflammation and tissue damage [60, 61].

Additionally, vitamin D effects may counteract monoamine oxidase (MAO)-induced oxidative damage. Monoamine oxidases (MAO-A and MAO-B) are mitochondrial enzymes involved in neurotransmitter catabolism, generating hydrogen peroxide and aldehydes as ancillary by-products. Increased MAO expression/activity has been reported to occur in the setting of obesity and also, with ageing in the cardiovascular system [62, 63]. Vitamin D has been also shown to modulate MAO expression, decreasing oxidative stress in neural [64, 65] and cardiovascular tissues [12].

Modulation of nitric oxide synthase (NOS) isoforms by 1,25(OH)_2_D_3_ observed in our study aligns with previous findings suggesting that vitamin D plays a significant role in regulating vascular homeostasis and reducing oxidative stress [14, 66, 67]. Specifically, we found that acute incubation with the active form of vitamin D significantly upregulated the gene expression of endothelial (eNOS) and neuronal (nNOS) isoforms while concurrently downregulating inducible NOS (iNOS) expression. This pattern of regulation suggests a shift toward more favourable nitric oxide (NO) production, as eNOS and nNOS are typically involved in the generation of low, physiological levels of NO, which play key roles in maintaining vascular tone, blood flow, and neuronal function [68, 69]. In contrast, iNOS is typically upregulated in inflammatory states and produces high levels of NO, which are associated with oxidative stress and tissue damage [70]. It is tempting to speculate that downregulation of iNOS expression observed in our study could also reduce the generation of reactive nitrogen species (RNS); of note, mitigation of neuronal nitrosative stress in response to acute incubation with vitamin D was reported in the literature in cell culture experiments [71]. Nevertheless, vitamin D exert protective effects against oxidative damage also via the modulation of NOS isoforms in the venous walls.

Overall, vitamin D exhibits a multifaceted role in controlling oxidative stress and may influence venous health beyond its classical roles in calcium and phosphate metabolism. The above described antioxidant effect requires further investigation since chronic venous disease, as atherosclerosis share vascular oxidative stress as a common pathophysiological mechanism. As such, these results should be regarded as the starting point for further mechanistic studies aimed at elucidating the signal transduction of vitamin D antioxidant effects in human venous samples. It has been reported in the literature that 1,25(OH)2D3 alleviated high-glucose-induced oxidative stress in human umbilical vein endothelial cells (HUVEC) via the upregulation of the nuclear factor erythroid 2-related factor 2 (Nrf2) antioxidant signalling pathway in a VDR-dependent manner; inhibition of Nrf2 nuclear translocation via siRNA abolished the antioxidant effect of vitamin D [72]. A similar result (lack of 1,25(OH)2D3 beneficial effect) was obtained with VDR expression inhibition with siRNA in an elegant study that assessed (among other) the beneficial effects of vitamin D on dextrose-induced oxidative stress in HUVEC [73].

Nowadays, chronic vitamin D supplementation has become rather controversial since several large intervention trials have not shown significant benefits in the elderly population with cardiovascular diseases (recently reviewed in Ref. [74]). The present pilot study showed that vitamin D exert protective effects in venous pathology that may be beneficial in acute administration prior to the surgical intervention. Understanding the molecular mechanisms underlying the interaction among vitamin D, obesity and CVD may provide novel insights into targeted interventions aimed at mitigating venous dysfunction and improving vascular health in patients with varicose veins.

### Limitations of the study

Despite the valuable insights provided by this pilot study, several limitations must be acknowledged. Firstly, we did not assess the expression/activity of the antioxidant enzymes that might have been acutely modulated by vitamin D in the venous samples. Second, while mRNA expression of NOS isoforms was assessed, protein expression was not measured, which may have provided complementary data on the acute effects of vitamin D on NOS modulation. Third, assessment of the inflammatory status in the clinical arena needs to be thoroughly assessed by measuring the levels of various pro-inflammatory cytokines (not only of CRP). Last but not least, all participants had low plasma vitamin D levels, which restricts the ability to draw conclusions about the effects of vitamin D in individuals with normal levels. Obviously, there is a need for prospective, randomized intervention studies in order to assess the optimal dosage, duration, and timing of vitamin D administration in patients with CVD who require surgical intervention.

## Conclusions

In the present study we have shown the role of vitamin D in the pathophysiology of varicose veins, by mitigating oxidative stress and modulating the expression of nitric oxide synthase isoforms. Additional research is needed to explore whether vitamin D supplementation or VDR activation have therapeutic potential in preventing and/or managing the varicose vein progression. Large clinical trials are required to recapitulate in the clinical arena the beneficial effects of acute vitamin D administration in patients with chronic venous disease with surgical indication.

## Data Availability

The authors confirm that data supporting the findings of this study are included within the article. No datasets were generated or analysed during the current study.
